# The risk of misclassifying subjects within principal component based asset index

**DOI:** 10.1186/1742-7622-11-6

**Published:** 2014-06-18

**Authors:** MA Yushuf Sharker, Mohammed Nasser, Jaynal Abedin, Benjamin F Arnold, Stephen P Luby

**Affiliations:** 1 icddr,b, 68 Shahid Tajuddin Ahamed Sarani, Mohakhali, 1212 Dhaka, Bangladesh; 2 Department of Statistics, University of Rajshahi, 6205 Rajshahi, Bangladesh; 3 School of Public Health, University of California, Berkeley, USA; 4 Global Disease Detection Branch, Division of Global Health Protection, Center for Global Health, Centers for Disease Control and Prevention, Georgia, USA

**Keywords:** Principal component analysis, Socio-economic status, Asset index, Wealth index

## Abstract

The asset index is often used as a measure of socioeconomic status in empirical research as an explanatory variable or to control confounding. Principal component analysis (PCA) is frequently used to create the asset index. We conducted a simulation study to explore how accurately the principal component based asset index reflects the study subjects’ actual poverty level, when the actual poverty level is generated by a simple factor analytic model. In the simulation study using the PC-based asset index, only 1% to 4% of subjects preserved their real position in a quintile scale of assets; between 44% to 82% of subjects were misclassified into the wrong asset quintile. If the PC-based asset index explained less than 30% of the total variance in the component variables, then we consistently observed more than 50% misclassification across quintiles of the index. The frequency of misclassification suggests that the PC-based asset index may not provide a valid measure of poverty level and should be used cautiously as a measure of socioeconomic status.

## Introduction

Socioeconomic status (SES) is commonly measured in social science and public health research by combining diverse factors including wealth, education level and occupation [1]. The asset index, which has also been called the wealth index, is created by measuring an individual’s assets and is widely used as a proxy measure of socioeconomic status. However, the asset index is a measure of the acquired assets and, as such, is a subset of total socioeconomic status. Nevertheless, public health researchers commonly use the asset index in regression models to either estimate its direct effect on outcomes or control for confounding effect on disease-exposure association [2-4].

To derive the asset index, researchers commonly gather information on asset ownership usually through the administration of a questionnaire and then frequently apply principal component analysis (PCA), as a data compression technique. The PCA method generates as many principal components as there are variables in the dataset. The first principal component (PC) is a weighted sum of the observed asset variables that accounts for the maximum variability of the observed data among other principal components. This first PC is considered as an asset index [5]. PCA allows the analyst to replace various asset variables with the univariate first PC score that best assigns the subjects into different categories. Subjects may then be classified into quintiles according to their asset index. For example, the first quintile, consisting of the lowest 20% values of the index, represents persons with the fewest assets (the poorest subject category) and the fifth quintile, consisting of the highest 20% index values, represents persons with the most assets (the wealthiest category).

Conceptually, there is a “true” measure of socioeconomic status which can not be determined and is associated with various outcomes, for example, a specific health outcome. Since we can not determine the true measure of socioeconomic status, we measure either related proxy variables, such as income, or manifest variables, such as presence of assets. Economic proxy and manifest variables are assumed to represent a person’s true economic status. When proxy variables are not available, researchers may use an asset index derived using PCA [5-8]. It is expected that the asset index would retain the order of the “true” socioeconomic status of the study subjects with negligible error and that it would provide the real quintile membership of a subject. There are, however, no standard tools available to validate the performance of the asset index, including the PC-based asset index, in terms of retaining the order of the true socioeconomic status of an individual because the underlying true socioeconomic status is unknown.

Several authors who have applied the PC-based asset index, have attempted to validate its credibility in different ways [5,6,9,10]. These studies reported satisfactory performance of PCA-based asset indices for explaining the variability of the fertility rate of a country, educational outcomes (schooling of children, drop out of children from schools) and income or expenditure based inequality measures.

Howe et al. compared four different methods to measure an asset index, including applying PCA on all categories of asset variables and applying PCA on binary coded asset variables. Using the data from the 2004 - 2005 Malawi Integrated Household Survey, they found that PC had modest agreement with consumption expenditure (kappa = 0.11 and 0.10) which is an intensive measure of household wealth used by economists as the optimal measure to assess income and welfare [11]. Howe et al. also reported the proportion of subjects that were misclassified by the PC-based asset index into the wrong asset quintiles when compared with consumption expenditure: 71% for all categories of asset variables, and 70% for binary coded asset variables. They concluded that a PC-based asset index was not a reliable proxy for consumption expenditure [12].

Kolenikov and Angeles used simulations to assess the performance of a PCA-based asset index for ranking the subjects compared to simulated welfare. They reported that the PCA-based asset index misclassified subjects into the wrong asset quintiles when compared to welfare quintiles, but did not explore the reasons behind the misclassification [10].

Howe et al. performed a systematic review of 17 articles with 36 datasets to see how the PC-based asset index performed compared to consumption expenditure and found that most of the asset indices poorly reflected consumption expenditure. The study considered different measures of asset indices in addition to PC-based asset indices but did not focus on reasons for poor performance of the asset indices [7].

In published literature of asset index measurements using PCA, the proportion of explained variances by the first PC were low, ranging from 12% to 34% [2,3,5,8,13]. Since researchers replace multiple asset variables by the single first PC score, a higher proportion of explained variance by the first PC is important to carry enough information of multiple asset variables [14]. However, we have little information about how the proportion of explained variance by the first PC could affect the performance of the PC-based asset index.

The use of misclassified covariates to control confounding can bias the exposure-disease association estimates [15,16]. If a PC-based asset index does not properly categorize study subjects when compared to real measures of wealth, it may be that the PC-based asset index may not be a good index to control confounding in exposure-disease association analyses. The objectives of this study were to verify whether a PC-based asset index would yield the same quintile rank as the quintile rank that was artificially imposed in the simulated data. This study also explored the possible reasons why PCA might perform poorly for asset index measurement.

## Methods

**Figure 1:**
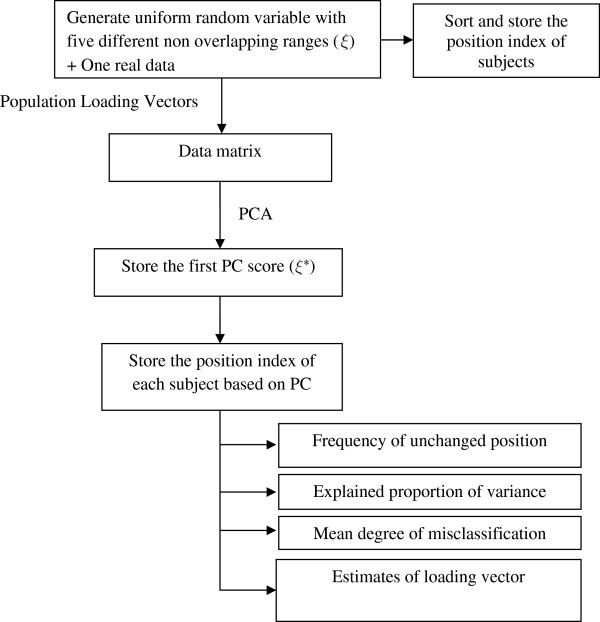
Flowchart of the simulation.

In this study, we performed a simulation experiment. In each simulation, we generated 100 random numbers from the uniform distribution of five different non-overlapping ranges as a measure of asset index. This simulated asset index was considered the true asset index of a group of 100 subjects. We then generated the asset variables using pre-specified loadings and the simulated index through a confirmatory factor model as described in Kolenikov and Angeles [10]. A confirmatory factor model extracts variables by taking proportions of the index plus the random error which is usually the measurement error. We used four sets of loadings for generating data that yielded four models. The process for generating the data and simulations are described in Table 1 and Figure 1. We wrote a customized program in *R* to perform the simulation experiment [17]. The program was tested by another co-author to check the reproducibility of the results.

**Table 1:** Data generating process in the simulation

	
●	We generated artificial latent factor *ξ* which is assumed to be the real asset index. We used *ξ* of 100 data points from the uniform distribution with five different arbitrary non-overlapping ranges, including (0,3], (3, 5] (5,8] (8, 10] and (10, 14]. We drew 20 sample points from each range and stored the position index of each subject based on the latent factor.
●	We considered normalized loading vectors *V*_1_=(0.79,0.54,0.13, 0.01,0.26), *V*_2_=(0.73,0.52,−0.20,0.00,−0.4), *V*_3_=(0.67,0.4,−0.5, −0.01,−0.4) and *V*_4_=(−0.02,0.14,−0.57,−0.51,−0.63).
●	We generated the data matrix *Y* based on the confirmatory factor analysis model used in Kolenikov and Angeles [10].
●	We generated five dimensional random variables using the loading vectors and standard normal errors *δ* with mean 0 and variance from (0,4].
●	We performed PCA on *Y* and generate the asset index *ξ*^∗^.

We then tested the performance of a PC-based asset index without any specific distributional assumption by using a real measurement of expenditure data, collected from an intensive qualitative survey, that was a skewed proxy measure of the economic status. We performed a similar experiment using the expenditure data instead of simulated asset index. The only difference between the simulated index and expenditure data was that in the simulation experiment, we generated a different asset index for each set of loadings, however in the expenditure data the asset index was fixed. We generated the artificial asset variables using the observed expenditure data and the same weights used in the simulated asset index. To make the results comparable with the units of the simulated asset index, we standardized the expenditure data using $\xi =\frac{x-\text{median}(x)}{\text{MAD}(x)}$ where *MAD*(*x*) is the median absolute deviation about median of the variable *x*. This standardized measurement was considered to be the true index for our experiment. We used the median for standardization to keep the position index of the subjects the same as in the original rank according to the expenditure data.

We repeated the experiment 10,000 times for each of the four models for a total of 40,000 replications for both the simulated index and the real expenditure data. If the PC score *ξ*^∗^ retained the order of *ξ*, which was the true index generated by the models, the position of an observation in *ξ* and *ξ*^∗^ should be the same. We recorded the frequency of the same position index which we defined as the frequency of unchanged positions.

We estimated the mean degree of misclassification in the PC-based asset index that involved classification of *ξ* and *ξ*^∗^ into their quintiles and counted the number of observations where the quintile membership was different. The probability of misclassification was estimated by dividing the total number of observations classified into different quintiles by the total number of observations in *ξ*.

We stored the proportion of explained variance by the first PC and estimates of the loadings for each of the replications. We explored the dependency pattern among the frequency of unchanged position, probability of misclassification, and explained proportion of variance using scatter plots. To assess the effect that the five different loadings estimate on the relationship between the proportion of explained variance and asset quintile misclassification, we constructed a parallel coordinate plot. In a parallel coordinate plot, the estimate of a loading vector that consisted of five elements was plotted into the five parallel vertical coordinates (E1-E5) and the plotted points were connected horizontally. Each connected line corresponded to a simulation result of loading vector estimates. Finally, we used different colors for the two clusters and identified the characteristics of loading estimates between the two clusters.

## Results

**Figure 2:**
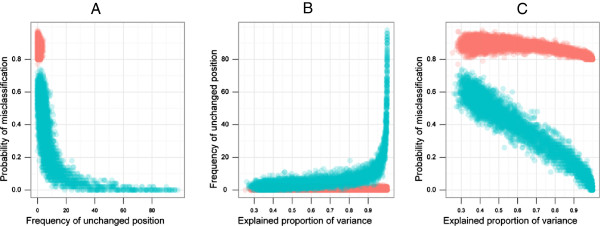
**Scatter plots between frequency of unchanged position and probability of misclassification (A), proportion of explained variance and frequency of unchanged position (B) and proportion of explained variance and probability of misclassification (C).** The data in red refer to those simulations where the probability of misclassification were consistently more than 80% irrespective of different levels of explained proportion of variance. The data in green refer to those instances where the probability of misclassification is negatively correlated with explained proportion of variance.

Our simulation study showed a range of 0% to 98% misclassification in the PC-based asset quintile. The median probability of misclassification varied from 44% to 82% depending on the different loading vector used for data generation. (Table 2). Because of the definition of the unchanged position and asset quintile misclassification, fewer than 20% of the unchanged positions produced up to 100% asset quintile misclassification. However, if the percent of unchanged position was greater than 20%, the mean degree of misclassification reduced dramatically (Figure 2A).

**Table 2:** Descriptive statistics of the number of unchanged order, and probability of misclassification into the wrong quintile for four different vectors in simulated data

	**Number of** **unchanged position**				**Maximum positive** **dispersion of position**				**Probability of** **misclassification**			
	***V***_**1**_	***V***_**2**_	***V***_**3**_	***V***_**4**_	***V***_**1**_	***V***_**2**_	***V***_**3**_	***V***_**4**_	***V***_**1**_	***V***_**2**_	***V***_**3**_	***V***_**4**_
Minimum	0	0	0	0	1	1	1	5	0	0	0	.04
First quartile	2	1	0	0	20	24	38	42	.25	.30	.46	.49
Median	4	3	1	1	37	44	93	76	.44	.50	.82	.67
Mean	7	6	4	2	38	50	68	69	.41	.51	.65	.66
Third quartile	7	6	4	3	52	92	97	97	.55	.82	.89	.87
Maximum	98	98	98	26	99	99	99	99	.97	.98	.98	.97

The pattern of the probability of misclassification using real expenditure data was similar to the simulated asset index. The observed misclassification ranged between 0% to 96%. The median probability of misclassification varied from 68% to 79% (Table 3).

**Table 3:** Descriptive statistics of the number of unchanged order, and probability of misclassification into the wrong quintile for four different vectors for real expenditure data

	**Number of** **unchanged position**				**Maximum positive** **dispersion of position**^**∗**^				**Probability of** **misclassification**			
	***V***_**1**_	***V***_**2**_	***V***_**3**_	***V***_**4**_	***V***_**1**_	***V***_**2**_	***V***_**3**_	***V***_**4**_	***V***_**1**_	***V***_**2**_	***V***_**3**_	***V***_**4**_
Minimum	0	0	0	0	2	2	1	13	0	0	0	.10
First quartile	1	1	0	0	56	63	73	71	.55	.59	.67	.65
Median	2	2	1	1	76	81	88	86	.68	.72	.79	.76
Mean	4	3	2	2	70	74	80	80	.64	.68	.74	.73
Third quartile	5	4	3	3	89	93	96	95	.79	.84	.87	.86
Maximum	86	95	88	19	99	99	99	99	.96	.96	.97	.96

^*^Real expenditure data has 112 observation, so the dispersion of position were rescaled to 100.

**Figure 3:**
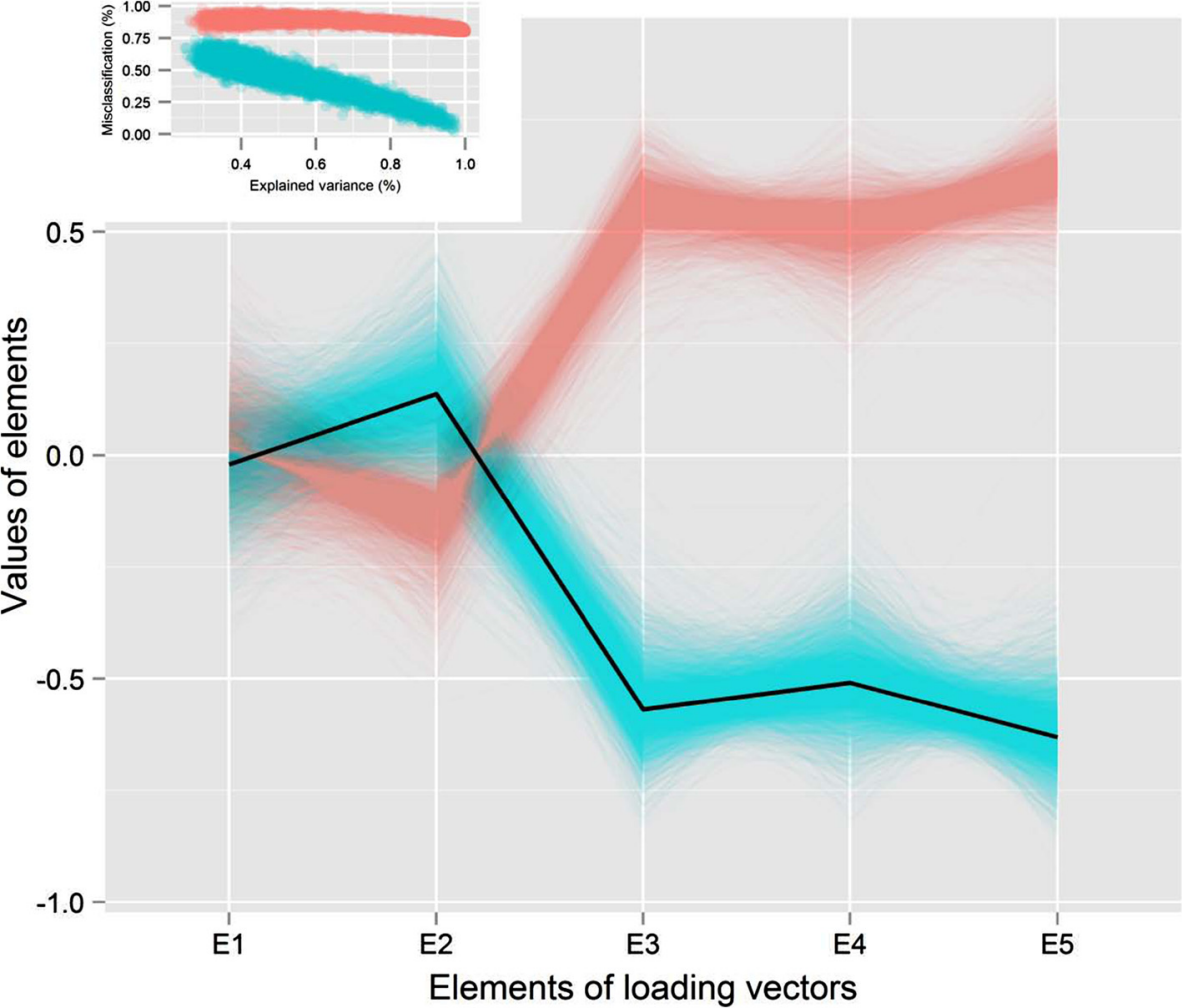
**Parallel coordinate plot of the elements of loading vectors.** The green color corresponds to the cluster that indicates the increasing proportion of explained variance decreases the percentage of misclassification into quintiles. The dark line indicates the population loading vector based on which data were generated. Inset, the scatter plot of the proportion of explained variance and percentage of misclassification matching color with the parallel coordinate plot corresponds which estimate of loading vectors are linked with those clusters.

The simulated data subjects were much more likely to retain their position when the first PC explained a large fraction (>90*%*) of the variance (Figure 2B). Iterations in which the PC explained close to 100% of the variance accounted for those instances in which up to 100% of subjects retained their position. However, there were a large number of subjects that did not retain their same position although their explained proportion of variance was even greater than 80% (marked as red points in the figure). (Figure 2B).The scatter plots between the proportion of explained variance and the probability of misclassification generated two clusters (Figure 2C). One cluster (marked as green) indicated that the mean degree of misclassification decreased with the increasing proportion of explained variance. The other cluster consistently showed more than 80% misclassification irrespective of the levels of the proportion of explained variance (marked as red) (Figure 2C).The parallel coordinate plot of the loading vector estimates in each experiment revealed that, the sign of the loadings has an important role in limiting the misclassification of the subjects. The red cluster included simulation results where the index consistently provided more than 80% misclassification. For those instances in the simulation, the signs of the loading estimates were found to be opposite from the real one (the sign of loading that were used for generating true index). In this situation despite the higher proportion of explained variance, PC-based index might not retain the original position of subjects. In the green cluster where the higher proportion of explained variance reduces the proportion of misclassification, we observed the retention of the signs of the loading estimates (Figure 3).

## Discussion

In this article we evaluated whether PCA retained the order of subjects based on a true asset index using a simulation experiment. We also used expenditure data collected in a different study to address the distributional limitations of the simulated asset index.

We found that PCA does not reliably maintain the order of the true asset index. PCA changes the position of up to 98% of subjects, and the magnitude of the position change was usually enough to classify the subjects into the wrong asset quintiles. We observed a relatively higher probability of misclassification when we considered observed expenditure data as a true index which was positively skewed. The skewed distribution of the underlying latent factor introduced more risk of the probability of misclassification in a PC based index. Our findings are supported by Kolenikov and Angeles [10] who reported the increased risk of misclassification in a PC-based asset index for those data which were generated from the skewed underlying factors.

In our simulations, the sign of the loading of the asset variables retained by the PCA was an important determinant of the probability of misclassification in a PC based asset quintile. A change in the sign means a change in the direction of contribution of an asset variable to the index. In the real world, an asset might positively contribute to relative wealth, but in the PC-based index, this might appear negatively. For example, the loading of agricultural land appeared with a negative sign in the PC-based asset index in Howe et al. [12]. The opposite sign may appear, possibly due to data coding and measurement scales selected and the underlying correlation structure within variables. Researchers often select asset variables in such a way that they are positively correlated to each other. While conducting the PC-based asset index among the positively correlated variables, the loading of those variables should appear with a positive signs. Our study suggests that loading variables might be assigned a negative sign because the underlying correlation among the asset variables might vary in different population. If so, the variables with negative loadings may be problematic because the presence of such an asset inappropriately leads a subject to the lower level from its true level in the index, and our simulations suggest that this could contribute importantly to misclassification [8,13].

The increased proportion of explained variance of the first PC score increases the probability of generating an index that reflects the underlying economic status. To ensure a higher proportion of explained variance of the dataset by the first PC, variables should be well correlated with each other. It is possible that asset variables might be classified into subgroups and/or might be redundant based on the correlation structure. When this occurs the first PC represents the subgroup of variables that contains the major source of variability of the total dataset and may not account for the contribution of all variables [14]. In such situations, only the first PC might not be sufficient either to account for the contribution of all asset variables or to explain a sufficient amount of variability required to reduce the misclassification of subjects. The situation becomes more difficult when asset variables are categorical. Proper variable selection and use of appropriate correlation for nominal and ordinal variables, such as polychoric correlations, could improve the power of explaining the variability of PC-based index [10].

To use PCA for an asset index, the sign of loadings should be examined in addition to the proportion of variance explained by the first PC in order to increase our confidence in the accuracy of the ranking of real wealth. The sign of the loading variables should be internally consistent with our understanding of what constitutes wealth of the study population. Additionally, checking consistency between wealth groups in respect to their existing asset variables and checking the robustness of the asset index with regards to different asset variables could help measure the level of reliability as was done by Filmer and Pritchett [5].

Although PC based asset index is a poor proxy against the standard consumption expenditure, it continues to be used because it is so much easier to deploy [7]. For example, after publishing the seminal paper of Filmer and Pritchett [5], we observed a couple of applications of PCA for estimating the asset index such as [2,3,6,8,13]; Some of these papers considered validity checking based on the correlation between the PC based index with some other proxy variables. If the correlation/association measurement approaches to 1, the order of the index approaches the order of the observed proxy. In addition to the correlation with some reliable proxies, the characteristics of the PCA based index such as loadings, sign of loadings and the proportion of explained variance should be reported as a tool for validation which were rarely considered to validate their indices of wealth.

To even engage in an exploration of possible algorithms applied to proxies of economic status, and examine those against a standard, implies an acceptance that the underlying data-generating distribution follows this model. Ideally, there would exist a measurable standard that we could compare algorithms applied to proxies and thus be able to argue for one approach versus another based on estimates of risk (e.g., probability of misclassification to which quintile a subject belongs). However, such a measurable standard does not exist for economic status. We have taken an approach that would identify which algorithms applied to proxies are best with regard to some loss function at predicting the latent variable under the best circumstances, where this sort of latent variable model is true. Thus the results should be interpreted knowing that the possible simulations (data-generating models) and possible methods for summarizing the manifest variables are but a tiny subset of the possible combinations. Our conclusions are meant to provide some intuition for problems that could arise, but can of course not be seen as proof by simulation.

Kolenikov and Angeles [10] showed that, heavy tailed distribution of SES index, such as a lognormal distribution, notably affects the coefficient estimates and the frequency of misclassification. They reported the marginal effect as 15% for overall misclassification and 30% misclassification in the first quintile in the PC-based procedures. All other distributions, including bimodal skewed distribution, have rather mild effects on misclassification. In this study, we only considered the asset index variables to be uniformly distributed. This limited the misclassification due to the distribution and allowed us to explore other contributors to misclassification. We measured the performance of the PCA method using data only with continuous variables. We expected that this would create fewer errors in datasets compared to a mix of continuous and categorical variables where there would be even greater misclassification using the PCA method [10]. Therefore, our estimates of misclassification are conservative. We considered the data matrix of only five dimensions. However, the results are still generalizable over higher dimensions because the rank preserving capacity of PCA in asset/wealth index should remain the same in higher dimensions. The work we present here could be expanded to use constructs simulated from actual asset variables in empirical datasets, which would be of higher dimension and include a mix of continuous and categorical asset variables.

Through repeated simulation experiments using artificial and real proxy data for latent variables, we showed that PCA does not retain the order of the true asset index and provides a high proportion of misclassification into the asset quintiles. Since the first PC score does not reliably maintain the original order of a latent construct, we should search for an alternative index that maintains the original order.

If investigators use PCA to create an asset index, they should report the proportion of variance explained and the loadings. Careful selection of asset variables, proper measurements and coding, and suitable correlation estimates of categorical asset variables are recommended to increase the variability explaining capacity of the first PC. If the proportion of explained variance is less than 30%, the risk of misclassification could be high (≥50*%*), so it should be interpreted with caution. We recommend checking for consistency and robustness for any level of explained variance. If the goal of the asset index is to control for confounding, then investigators should consider the asset variables as the original covariates in the model, which we expect (though have not tested) could more completely controlled for confounding than PC-based indices.

## Competing interests

The authors declare that they have no competing interests.

## Authors’ contributions

YS developed the simulation study design and drafted the manuscript, MN provided theoretical support in developing study design, JA provided input in programming, and data presentation, SL supervised the process. All the authors critically reviewed, provided intellectual input to the manuscript and approved the final version of the manuscript.
